# Effect of shearing on some physiological and hormonal parameters in Akkaraman sheep

**DOI:** 10.5713/ajas.19.0400

**Published:** 2019-10-21

**Authors:** Erkan Pehlivan, Mahmut Kaliber, Yusuf Konca, Gürsel Dellal

**Affiliations:** 1 Department of Animal Science, Faculty of Agriculture, Ankara University, Ankara 06110, Turkey; 2 Department of Animal Science, Faculty of Agriculture, Erciyes University, Kayseri 38039, Turkey

**Keywords:** Stress, Fleece, Thermoregulation, Climatic Factors, Homeostasis

## Abstract

**Objective:**

Shearing is one of the practices that is applied periodically to fiber producing animals, which can also alter resistance of animals to high temperatures in especially summer months. This study aimed to investigate effects of shearing on some physiological and hormonal parameters in Akkaraman sheep during summer season.

**Methods:**

This study was carried out on 39 non-pregnant Akkaraman ewes (aged 1.5 years at the beginning of experiment). The 39 ewes were chosen randomly from the flock belonging to the Erciyes University and they were assigned to two groups as follows: i) group A (n = 20) designed as the control group, they were shorn and group B (n = 19) designed as the experimental group, they were unshorn. Prior to the shearing (−1 day) and on days 1, 7, 15, 30, 45, 60, 75, and 90 following the shearing, blood samples were taken from the vena jugularis of each sheep. Cortisol, β-endorphin, growth hormone (GH), thyroxine (T_4_), triiodothyronine (T_3_), and heat shock protein 70 (HSP-70) concentrations were determined using the enzyme immunoassay method. Body weight (BW), rectal temperature (RT), pulse rate (PR), and respiratory rate (RR) of each sheep were recorded at the same time. The data obtained were analyzed using two-way repeated measures analysis of variance.

**Results:**

Statistical analysis showed a significant effect of shearing×period interaction (p< 0.01) and a significant effect of period (p<0.01) on BW, HSP-70, cortisol, T_4_ and RT, PR, GH, β-endorphin, T_3_, respectively. Also these analysis showed no significant effect of shearing× period interaction or period on RR.

**Conclusion:**

The results showed that the thermoregulation abilities of sheep were affected by shearing treatment and the shorn ewes were less affected by heat stress. In conclusion, based on the data of this study, shearing can be considered as a necessary management practice that requires protection for sheep from the effect of heat stress.

## INTRODUCTION

There are many different practices such as tagging, tail docking, dehorning, castration, weaning, vaccination, bathing, hoof trimming and shearing in sheep husbandry. Some of these practices are performed only once in the animal’s lifespan, while others are necessarily repeated periodically and these may cause an endocrine and metabolic response to occur in animals known as stress [1,2]. Animals that are exposed to stress react in species-specific behavioral patterns that are also influenced by learned behavior, and there are differences between animals in response to these behaviors. Animals, depending on their genetic structure and previous experience, can respond differently to the same stressors. Moreover age, gender, physiological status, herd density, daily rhythm and other environmental factors can also affect the individual reactions that animals show against the stressors [3,4]. Under different environment and management conditions, animals that are exposed to stressors continue their existence and production through behavioral responses, metabolic changes that occur in different biological systems, along with physiological parameters such as heart rate, respiration rate and as well as body temperature. During the stress, maintenance of the homeostasis of the animal is carried out essentially through the control of the physiological processes, mainly cortisol and other stress hormones as well as metabolic hormones such as thyroxine (T_4_), triiodothyronine (T_3_), insulin-like growth factor-1, growth hormone (GH), and β-endorphin which also play role in these control processes [4,5]. By monitoring, measuring and evaluating the changes in these biological parameters, it is possible to decide on the type, stage and precautions to take against stress [3,4,6].

Occasionally, different stress factors can also be effective in sheep breeding. While some of these stress factors are mainly due to inadequate and/or poor feed and water resources, climatic factors such as high temperature and humidity may also be effective especially in some periods. Fleece plays an important role in the response of sheep to these climatic factors. The fleece has a natural thermoregulatory structure and it provides thermal insulation which reduces convective heat loss from the body under cold environment conditions and radiative heat gain from the environment under hot environment conditions [7,8]. In addition, fleece is an economic product which has a significant share among the breeders’ incomes. Therefore, although it varies according to the countries, the shearing is usually done once a year to harvest the wool of sheep [9]. As in many European countries [9], traditionally shearing time in Turkey is the spring and early summer seasons that coincide with temperate climates. The shearing is considered not only for hygienic reasons but also as a compulsory practice to improve the sheep’s’ resistance to high temperatures in summer [10]. During shearing, animals’ welfare is adversely affected, and short-term acute stress occurs [11–13]. The acute stress that occurs during the shearing is not only due to the shearing, but also from the preparation procedures such as capture, separation, immobilization and tying required for the shearing. However, this traditional practice also means harvesting the fleece which provides protection against the external factors. In some of the studies performed in this field, it was reported that shearing increased the sensitivity of the animals to thermal stress [8,10–15], in others [2,16,17], that the removal of fleece did not have a significant effect on the physiology of the sheep or that sheep were becoming more resistant to thermal stress after shearing. However, fleece properties of sheep have a very important role in the response of sheep to thermal stress [18].

From this point of view, this study was aimed to determine both effect of shearing and the reactions against climatic factors after shearing on some physiological and hormonal parameters in young Akkaraman ewes that not previously been sheared.

## MATERIALS AND METHODS

### Animal care

The experimental procedure was approved by Local Ethics Committee at Erciyes University (16/004).

### Location, experimental animals, shearing procedures, and experimental groups

This study was carried out between June and September months of 2016 in a farm entitled Erciyes University Agricultural Research and Application Centre (ERUTAM) located in Kayseri, Turkey (38°29′21.5″ N; 35°10′11.7″ E) at an altitude of approximately 1,130 meters above sea level. The study was conducted on 1,5 years old 39 non-pregnant Akkaraman ewes at the beginning of the experiment. The 39 ewes were chosen randomly from the flock belonging to the Erciyes University. During 15 days prior to starting of the study, all ewes were identified and subjected to internal and external parasite controls. The following experimental protocol was used: the ewes were randomly divided into two groups; i) Group A (n = 20) designed as the control group, and were shorn and group B (n = 19) designed as the experimental group, and were unshorn. The shearing was performed via sheep shearing machine (Xpert 708-200, Heiniger AG, Herzogenbuchsee, Switzerland) 11:00 am and 2:00 pm on the same day, without any interruption. All the ewes were clinically healthy. During the experimental period, the ewes grazed on natural pasture between 7:00–10:00 am and between 4:00–07:00 pm and they were housed in shaded pens (2 m^2^ per sheep) in the remaining time and natural light from windows and a door could pass through to the pens. The ewes were fed with 400 g concentrated commercial feed and 400 g alfalfa hay per head as a supplement in every day during this period and they were not milking. Fresh water was always available to the ewes. The study was conducted within standard ethical norms.

### Blood collection and determination of body weight and some physiological parameters

Blood samples (8 mL) were taken regularly from the vena jugularis of each ewe into vacuum containers without any anticoagulant (VACUETTE TUBE 8 mL Z Serum Sep Clot Activator, Greiner Bio-One, Kremsmünster, Austria) on experimental days before shearing (−1 day) and after shearing (1 day) and were repeated after 7, 15, 30, 45, 60, 75, and 90 days from the shearing date. The blood samples randomly taken from the 8 ewes in each group (group A, n = 8, group B, n = 8) within 1 hour (1:00 pm and 2:00 pm) were subjected to hormone analysis. Samples were centrifuged at 3,000 rpm for 10 min and the serum was separated and stored at −20°C until analysis time. Body weight (BW), pulse rate (PR), respiratory rate (RR), and rectal temperatures (RT) of each ewe were determined on the same times (2:00 pm and 4:00 pm) of same days as the blood collection days. The BW were determined using a digital scale (±100 g, Beko, TEM Terazi, İstanbul, Turkey). The RR and PR were measured using a stethoscope (Classic III, 3M Littmann, St. Paul, MN, USA). The RT was measured with a digital thermometer (FTC-77030, Medisana, Neuss, Germany) with the probe being inserted into the ewes’ rectum to a depth of 4 cm.

### Hormone analysis

Analysis of hormones in the blood serum were performed by enzyme immunoassay method using microplate washer (W206, EMP Medical, Shenzhen, Guangdong, China) and microplate reader (M201, EMP Medical, China) in the Laboratory of Animal Physiology and Endocrinology at Erciyes University, Faculty of Agriculture, Department of Animal Science. The GH (Sunred, 201-07-0075, Shanghai, China), heat shock protein 70 (HSP-70; Sunred, 201-07-1139, China), β-endorphin (Sunred, 201-07-0095, China), cortisol (Sunred, 201-07-0067, China), T_3_ (Sunred, 201-07-1022, China), and T_4_ (Sunred, 201-07-1026, China) concentrations were determined using species-specific commercial enzyme linked immunosorbent assay kits. The minimum detectable concentrations of the kits were 0.066 ng/mL, 0.46 ng/mL, 4.827 ng/L, 0.183 ng/mL, 0.242 nmol/L, and 9.005 nmol/L for GH, HSP-70, β-endorphin, cortisol, T_3_, and T_4_, respectively.

### Climatic values

Climatic values on the location of the farm where the experiment was carried out, were obtained from Turkish State Meteorological Service [19] in order to estimate the severity of heat stress during the experimental period. Temperature-humidity index (THI) were calculated using the equation below reported by Marai et al [20] for sheep and goats [21].

THI=db °C-[(0.31-0.31 RH/100) (db °C-14.4)]

where db °C is the dry bulb temperature (°C) and RH is the relative humidity (RH %)/100. The values obtained indicate the following: <22.2, absence of heat stress; 22.2 to <23.3, moderate heat stress; 23.3 to <25.6, severe heat stress; and 25.6 and more, extreme severe heat stress [21].

### Statistical analysis

The data obtained from the experiment were analyzed using two-way repeated measures analysis of variance (ANOVA). Sources of variation were group (A and B), period (−1, +1, +7, +15, +30, +45, +60, +75, +90) and their interactions. Duncan multiple comparison test was performed to determine significant differences between groups and periods. The IBM SPSS STATISTICS 20 software package was used to perform ANOVA and MSTAT-C statistical software was used to Duncan multiple comparison tests [22].

## RESULTS

The values of average environmental temperature, relative humidity levels and THI on the sampling days are presented in Table 1. As can be seen from Table 1, values of average temperature and THI increased substantially in the second half of the experiment and the ewes were affected by severe heat stress on the 45th and 60th days of the experiment and moderate heat stress on the 90th day.

The average values of BW, RT, PR, RR, and GH, HSP-70, β-endorphin, cortisol, T_3_, and T_4_ in the groups during the experiment are presented in Table 2 and 3, respectively. Statistical analysis showed a significant effect of shearing×period interaction (p<0.01) and a significant effect of period (p<0.01) on BW, HSP-70, cortisol, T_4_, and RT, PR, GH, β-endorphin, T_3_, respectively. Also, these analysis showed no significant effect of shearing×period interaction or period on RR.

## DISCUSSION

Carcangiu et al [1] reported that not only the shearing, but also the preparation procedures caused acute stress in sheep. In this study, no significant difference was found on the obtained values (except BW and T4) between before shearing and 1 day after shearing (Tables 1, 2). This may be due to the first sampling being on 1st day after shearing. So indeed, acute stress is a process that develops very rapidly and disappears a short time after the stressor disappears [6]. It has been reported in the studies conducted on this field that cortisol hormone started to decrease towards basal level with the disappearance of acute stressors in ewes [23–25]. As a result, shearing (including preparation procedures for shearing) causes acute stress but the ewes rapidly regain their body homeostasis with the disappearance of these stressors.

Although shearing is a management practice causing acute stress, it can also alter the thermal homeostasis of the ewes. Because, thermal regulation in ewes is mainly influenced by their fleece properties among other factors [18]. In addition, climatic factors also play an important role in thermal homeostasis of sheep together with endogenous factors such as breed, age, gender and physiological status [10,15,16]. Climatic factors include thermal irradiation and wind speed as well as THI are evaluated together to determine the intensity of heat stress [15,21]. In the present study, THI values reached levels that will generate heat stress conditions after the 30th day of the experimental period. The ewes were affected by severe heat stress on the 45th and 60th days of experiment and moderate heat stress on the 90th day (Table 1).

**Figure 1:**
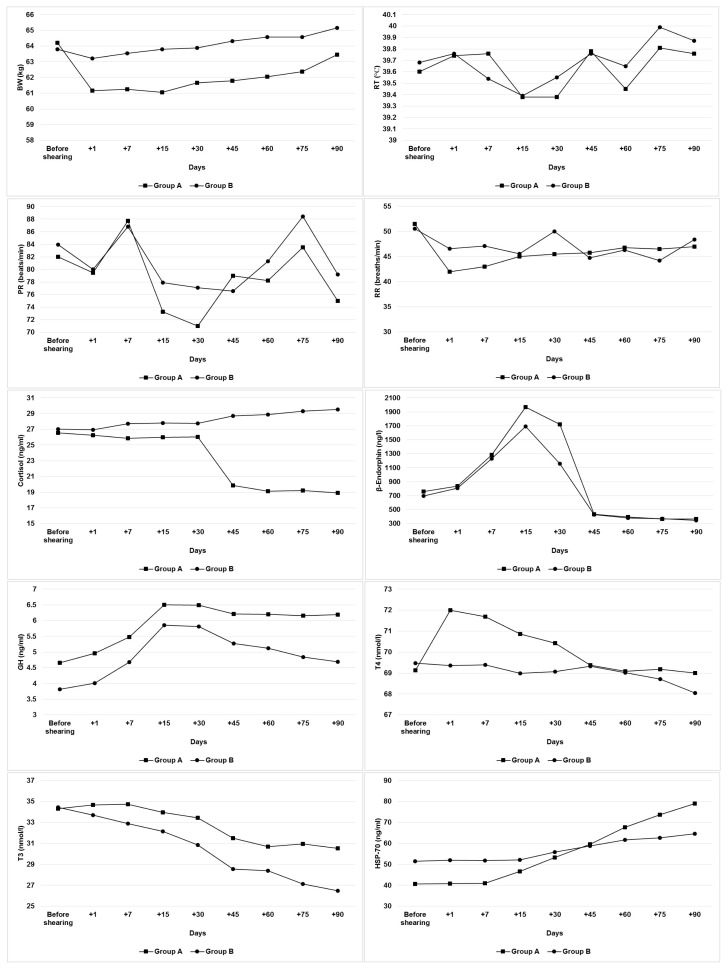
The patterns of BW, RT, PR, RR, cortisol, β-endorphin, GH, T_4_, T_3_, and HSP-70 in both groups during the experiment. Group A, shorn group; Group B unshorn group; BW, body weight; RT, rectal temperature; PR, pulse rate; RR, respiratory rate; GH, growth hormone; T_4_, thyroxine; T_3_, triiodothyronine; HSP-70, heat shock protein 70. Sampling was before shearing (−1 day) and on days +1, +7, +15, +30, +45, +60, +75, and +90 following the shearing.

The RT, PR, and RR as physiological responses are con sidered as indicators of the degree of thermal stress [26]. In this study, no significant difference was found between the groups in terms of these physiological parameters. However, the effect of period on RT and PR was significant (p<0.01) (Table 2) and the RT and PR of sheep reached higher levels in periods of heat stress than other periods (Figure 1). In fact, when animals are exposed to thermal stress, RT, RR, and PR are increased [27]. However, in this study, the RT and PR of the shorn ewes (group A) were generally lower than the unshorn ewes (group B). It can be said that this is due to the better adjustment of the heat distribution of the shorn animals, because fleece is an important factor affecting heat distribution in the body [9]. Fleece inhibits evaporation of water from the body reducing heat loss through sweating [28]. For this reason, the removal of fleece facilitates the adaptation of the ewes to high temperatures. It has been reported that at high ambient temperatures, shorn ewes dispersed 50% of body heat by means of evaporation better than unshorn ewes [29]. These findings were consistent with the findings obtained from previous studies [14,16,17,30,31] conducted in different sheep breeds.

Hormonal signal system plays a vital role in regulating homeostasis in organisms [4]. The main hormones in coping with stress are glucocorticoids and catecholamines. Changes in the concentrations of these hormones are indicative of adrenal activity [32]. In this study, the cortisol concentrations increased in parallel with the increase in environmental temperature in the unshorn ewes (group B) and reached the highest levels after with the onset of heat stress. The release of cortisol during stress varies according to the type and intensity of stress [4]. Thus, cortisol contributes to the preservation of homeostasis by regulating energy metabolism during stress. Adverse environmental conditions cause an increase in the secretion of the cortisol hormone in organism [33]. On the other hand, there was a significant decrease (p<0.01) of cortisol concentrations in the shorn ewes during the same period in this study. Apparently, these ewes were not intensely affected by heat stress due to the removal of their fleece. Therefore, this was due to the lowering of cortisol levels in order to decrease metabolic rate due to increase of temperature. β-Endorphin is a compound that plays a role in stress response like cortisol [34]. In this study, β-endorphin concentrations were found to be high until the onset of the heat stress in both groups and then it decreased suddenly and reached its lowest concentrations during the periods of heat stress. Although we could not find other studies to compare these values obtained during heat stress in sheep, it may be that this decrease in β-endorphin concentrations was related to a negative feedback mechanism of cortisol. In addition to cortisol, metabolic hormones such as GH, T_4_, and T_3_ are also affected by stress and therefore, it is reported that changes in the concentrations of these hormones may be an indicative assessment of animal welfare [4,5]. Although GH is one of the hormones involved in stress response, changes in GH concentrations during stress vary between species [1]. In this study, GH concentrations were increased until the onset of the heat stress periods in both groups and then decreased (p<0.01) during the heat stress periods and this decrease was more severe in the unshorn group (group B). As a matter of fact, GH concentrations decrease significantly depending on the type and severity of stress during long-term stress conditions [4,35]. Therefore, it can be said that this decrease in GH concentrations was caused by the decrease in the anabolic activities of organism due to the presence of stress. Similar results were also observed in bovines [36]. Todini [37] reported that systemic functions of thyroid hormones play a crucial role in the mechanisms enabling the adaptation of the animals to the environment and these hormones levels in circulation may decrease depending on high temperature and increase depending on low temperature. In this study T_4_ and T_3_ concentrations also decreased significantly due to increase of temperature. However, this decrease was higher in the unshorn group (group B). Besides, there was a significant increase in T_4_ and T_3_ concentrations in the shorn group immediately after the shearing (Figure 1). These increases in thyroid hormones may be caused by short-term stress. Acute physiological stress increases the secretion thyroid stimulating hormone from the pituitary gland and this increases the release of thyroid hormones from the thyroid gland to increase the metabolic rate [4].

The HSPs are a group of highly conserved proteins that are induced in both prokaryotes and eukaryotes by elevated temperatures or a variety of cellular stresses [38]. The HSP-70 has been most consistently associated with protection against conditions involving oxidative stress in organism [35]. In this study, while HSP-70 concentrations generally followed a flat trend up to the periods of heat stress, they showed a significant increase in periods of heat stress (Figure 1). It has been reported that HSP-70 is strictly stress-inducible and can only be detected following a significant stress upon the cell and organisms [39].

## IMPLICATIONS

In this study, shearing caused short-term stress in shorn ewes but the ewes had rapidly regained their body homeostasis. In addition, the main effect of the shearing was observed during the periods of heat stress and shorn ewes were less affected from this stress. During the summer when sunlight comes at a right angle, shearing can be considered as a necessary management practice and the effect of heat stress can be reduced in sheep that are housed in shaded shelters.

## Figures and Tables

**Table 1:** Average temperature (°C), relative humidity (%), and THI during the experimental days

Days	Average temperature (°C)	Relative humidity (%)	THI
Before shearing	18.8	68.3	18.4
After shearing (+1)	17.1	73.3	16.9
After 7 days (+7)	20.3	59.5	19.6
After 15 days (+15)	18.5	61.9	18.0
After 30 days (+30)	23.3	51	21.9
After 45 days (+45)	26.1	28.8	23.5
After 60 days (+60)	26.6	40	24.3
After 75 days (+75)	20.2	47	19.2
After 90 days (+90)	24.7	43.3	22.9

THI, temperature-humidity index.

**Table 2:** Average values (±standard error) of body weight, rectal temperature, pulse rate, and respiratory rate in the groups during the experiment

Parameters	Group	Before shearing −1	Days after shearing							

			+1	+7	+15	+30	+45	+60	+75	+90
BW (kg)	A (n = 20)	64.20±1.49^A^	61.17±1.48^C^	61.25±1.40B^C^	61.05±1.41^C^	61.67±1.51^BC^	61.80±1.40^BC^	62.05±1.42^BC^	62.38±1.64^B^	63.45±1.50^A^
	B (n = 19)	63.79±1.10^BC^	63.21±1.04^C^	63.53±1.05B^C^	63.79±1.11^BC^	63.89±1.15^BC^	64.32±1.11ABC	64.58±1.13^AB^	64.58±1.17^AB^	65.16±1.04^A^
	Total (n = 39)	64.00±0.92	62.17±0.92	62.36±0.89	62.39±0.92	62.76±0.96	63.03±0.91	63.28±0.93	63.45±1.02	64.28±0.92
RT (°C)	A (n = 20)	39.60±0.07	39.74±0.09	39.76±0.07	39.38±0.08	39.38±0.08	39.78±0.09	39.45±0.05	39.81±0.08	39.76±0.08
	B (n = 19)	39.68±0.07	39.76±0.09	39.54±0.08	39.39±0.08	39.55±0.07	39.76±0.07	39.65±0.07	39.99±0.07	39.87±0.10
	Total (n = 39)	39.64±0.05^BC^	39.75±0.06^AB^	39.65±0.05^BC^	39.39±0.06^E^	39.46±0.05^DE^	39.77±0.06^AB^	39.55±0.04^C^^D^	39.90±0.05^A^	39.81±0.06^A^
PR (beats/min)	A (n = 20)	82.00±2.31	79.50±2.20	87.75±2.63	73.25±3.00	71.00±3.62	79.00±2.10	78.25±2.03	83.50±2.12	75.00±1.50
	B (n = 19)	83.95±2.69	80.00±2.76	86.84±3.11	77.89±3.27	77.11±3.49	76.58±4.14	81.32±2.35	88.42±2.62	79.21±2.07
	Total (n = 39)	82.95±1.75^AB^	79.74±1.73^BC^	87.31±2.00^A^	75.51±2.22^C^^D^	73.97±2.53^D^	77.82±2.26^BC^^D^	79.74±1.55^BC^	85.90±1.70^A^	77.05±1.30^C^^D^
RR (breaths/min)	A (n = 20)	51.50±2.38	42.00±2.06	43.00±2.52	45.00±2.29	45.50±1.53	45.75±2.30	46.75±3.11	46.50±1.41	47.00±2.06
	B (n = 19)	50.53±2.96	46.58±2.26	47.11±2.17	45.53±1.48	50.00±2.59	44.74±2.80	46.32±2.19	44.21±1.16	48.42±1.75
	Total (n = 39)	51.03±1.87	44.23±1.55	45.00±1.68	45.26±1.36	47.69±1.51	45.26±1.78	46.54±1.89	45.39±0.93	47.69±1.35

BW, body weight; RT, rectal temperature; PR, pulse rate; RR, respiratory rate.

A–C Mean values within a row with different capital letters differ significantly (p<0.01) in sample comparison.

**Table 3:** Average values (±standard error) of cortisol, β-endorphin, growth hormone, T_4_, T_3_, and HSP-70 in the groups during the experiment

Parameters	Group	Before shearing −1	Days after shearing							

			+1	+7	+15	+30	+45	+60	+75	+90
Cortisol (ng/mL)	A (n = 8)	26.57±6.54^A^	26.23±7.04^A^	25.88±6.84^A^	26.01±6.67^A^	26.05±7.10^A^	19.86±4.56^B^	19.13±4.28^B^^b^	19.23±4.46^B^^b^	18.93±4.38^B^^b^
	B (n = 8)	27.04±4.87^A^	26.96±4.83^A^	27.73±4.43^A^	27.82±4.35^A^	27.77±4.42^A^	28.69±4.52^A^	28.90±4.42^A^^a^	29.32±3.98^A^^a^	29.52±3.78^A^^a^
	Total (n = 16)	26.80±3.94	26.59±4.12	26.80±3.94	26.92±3.85	26.91±4.05	24.28±3.30	24.01±3.23	24.28±3.17	24.23±3.11
β-Endorphin (ng/L)	A (n = 8)	758.00±218.00	839.00±261.00	1,286.00±320.00	1,970.00±618.00	1,719.00±515.00	433.00±76.40	397.40±76.20	368.00±69.20	366.00±62.40
	B (n = 8)	695.50±40.40	806.50±87.70	1,231.00±209.00	1,693.00±305.00	1,159.00±155.00	431.20±54.60	382.30±45.70	372.60±37.50	349.20±42.90
	Total (n = 16)	727.00±107.00^CD^	823.00±133.00^C^	1,259.00±185.00^B^	1,832.00±335.00^A^	1,439.00±270.00^B^	432.10±45.40^D^	389.90±43.00^D^	370.30±38.00^D^	357.60±36.60^D^
GH (ng/mL)	A (n = 8)	4.66±1.20	4.96±1.23	5.48±1.48	6.50±1.42	6.49±1.45	6.21±1.33	6.20±1.23	6.16±1.17	6.19±0.95
	B (n = 8)	3.82±0.98	4.01±0.97	4.68±0.86	5.86±0.84	5.81±0.90	5.27±0.83	5.12±0.74	4.84±0.79	4.69±0.80
	Total (n = 16)	4.24±0.76^D^	4.49±0.77^CD^	5.08±0.83B^CD^	6.18±0.80^A^	6.15±0.83^A^	5.74±0.77^AB^	5.66±0.71^AB^	5.50±0.70^AB^	5.44±0.63ABC
T_4_ (nmol/L)	A (n = 8)	69.13±1.66^CD^	71.99±1.62^A^	71.69±1.60^AB^	70.87±1.85^AB^	70.42±1.54^BC^	69.38±1.74^CD^	69.09±1.73^CD^	69.18±2.06^CD^	69.00±1.92^D^
	B (n = 8)	69.47±1.19^A^	69.35±1.30^AB^	69.39±1.46^AB^	68.99±1.47^AB^	69.06±1.51^AB^	69.33±1.35^AB^	69.01±1.34^AB^	68.71±1.36^AB^	68.04±1.32^B^
	Total (n = 16)	69.30±0.99	70.67±1.06	70.54±1.09	69.93±1.17	69.74±1.06	69.36±1.07	69.05±1.06	68.94±1.20	68.52±1.13
T_3_ (nmol/L)	A (n = 8)	34.32±7.47	34.68±7.65	34.74±7.14	33.97±6.43	33.43±6.64	31.49±6.39	30.69±5.42	30.96±5.49	30.54±5.43
	B (n = 8)	34.43±6.46	33.71±5.85	32.89±5.45	32.13±5.81	30.85±6.57	28.54±5.98	28.39±6.04	27.13±5.79	26.49±6.05
	Total (n = 16)	34.37±4.77^A^	34.19±4.65^AB^	33.81±4.35^AB^	33.05±4.20^AB^	32.14±4.52^B^	30.01±4.24^C^	29.54±3.93^C^	29.05±3.89^C^	28.52±3.96^C^
HSP-70 (ng/mL)	A (n = 8)	40.62±8.96^E^	40.83±9.18^E^	40.96±9.30^E^	46.60±10.00^E^	53.20±10.90^D^	59.60±11.00^C^	67.60±10.30^B^	73.70±11.00^AB^	79.02±9.91^A^
	B (n = 8)	51.47±9.86^D^	51.90±10.00^CD^	51.73±9.84^CD^	52.19±9.91^CD^	55.87±9.71BCD	58.71±9.96ABC	61.60±11.00^AB^	62.70±10.70^AB^	64.50±11.10^A^
	Total (n = 16)	46.05±6.58	46.38±6.72	46.35±6.69	49.38±6.86	54.56±7.05	59.15±7.16	64.60±7.32	68.20±7.55	71.77±7.42

GH, growth hormone; T_4_, thyroxine; T_3_, triiodothyronine; HSP-70, heat shock protein 70.

A–E Mean values within a row with different capital letters differ significantly (p<0.01) in sample comparison.

a,b Mean values within a column with different lower-case symbol differ significantly (p<0.01) in group comparison.
